# Donation-Parties

**Published:** 1860-06

**Authors:** 


### HALL'S JOURNAL OF HEALTH.

**Our Legitimate Scope is almost boundless: for whatever begets pleasurable and harmless feelings, promotes Health; and whatever induces disagreeable sensations, engenders Disease.**

WE AIM TO SHOW HOW DISEASE MAY BE AVOIDED, AND THAT IT IS BEST, WHEN SICKNESS COMES, TO TAKE NO MEDICINE WITHOUT CONSULTING A PHYSICIAN.

**Vol. VII.]** **JUNE, 1860.** **[No. 6.**

### DONATION-PARTIES,

SURPRISE-visits, lottery-drawings, fairs, *et id omne*, away with them! they are a hypocrisy, a cheat, a degradation, the whole of them, not a single exception, and being so, the sooner they are abandoned the better.

Donation-parties and surprise-visits are the ways and means of giving material aid to clergymen, who either need it or do not: if they do not need this aid, then the proceedings are simply a stultification of all concerned; if they do need such aid, it shows the great inconsideration if not actual injustice, of those to whom the minister preaches; it clearly indicates the fact that he is not properly sustained, and that his parishioners know it.

The practical workings of these machineries are always deceptive, always degrading, and lead to unmixed harm; they are a pecuniary loss to the clergyman himself, and a moral loss to the people of his charge.

It is the nature of gifts to degrade, to cause a feeling of dependence, of inferiority, and of obligation. A minister's palm should be as guiltless of a bribe as that of a judge. No foreign minister of our government is allowed to receive a present of any description in his official capacity, or even privately, by virtue of his station; not even the President of the United States can receive a gift or present from any nation. This is wise, and is based on a true knowledge of human nature; and neither ought a minister of the Gospel, who is, by virtue of his office, a minister from the Court of the King of kings, an ambassador from the skies.

To the disgrace of the American people, three fourths of the clergy who should live by the Gospel, who should be amply supported, are not adequately paid, are compelled, if they have no private means, to the most pinching economies; live in circumstances distressingly straitened; and endured, too, in multitudes of cases, with an uncomplainingness and a heroic courage which is beyond praise.

The usual history of a case is this; a Society needs a minister; they engage to give him a certain amount, which is most generally barely if at all adequate to his necessities, and the people know it. Perhaps not one in any dozen would be willing to give a year's labor for what is promised the minister; but there is an unexpressed feeling that there are certain perquisites which may supply the deficiencies; there are the wedding-fees, and the donation-visits and surprise-parties, the proceeds of which are of course greatly exaggerated; the consequence is an actual pecuniary loss in the long run to the minister, who is supposed to have been benefited by them. If there were no perquisites at all, then a larger salary would have been provided, and it would be more promptly and punctually and fully paid, with the incalculable advantage of enabling him to make his calculations and frame his expenditures to the amount received, with the result of a mind at ease all the year round. But knowing the salary to be inadequate, and not knowing what the sum total of the perquisites will be, there is hesitancy, uncertainty, disquietude, perplexity and unrest from one year's end to another, crippling the energies of the minister himself, depressing his wife, and causing a somber cloud to rest upon the whole household, to the moral injury of the minister himself and that of every single member of his society. The most wearing of all feelings is that of uncertainty, and especially of apprehension, and these are the abiding feelings of a large class of men who are literally the salt of the moral world, the national conservators; men who, for intellectual attainments, for moral culture, and a refinement of feeling at once elevating and pure, have not their equals in all human society besides; and all the more galling to these superiorities, are the uncertainties of an indefinite salary.

Another constant source of deception is, presents of articles are made, which the minister's family does not want; or are commercially valuable, but not worth to the minister one tenth part of what they cost. A twenty dollar "work-box" is a beautiful present to a minister's wife; but she could do without it, and two dollars in money would be practically more valuable to her. That two dollars would purchase a religious newspaper, which would be of more actual advantage to a minister's growing family, and to himself, too, than a room full of twenty-dollar work-boxes, which, as presents, he would not feel at liberty to sell. Moral: If you have any thing to give to your minister, give it to him in money, and it will be worth to him at least double its value in any thing else, in three cases out of four.

It then follows that these machineries, which the Evil One no doubt contemplates the workings of with considerable satisfaction, operate a pecuniary loss, both to the receiver and to the giver; to the giver, because two dollars in money would have done more actual good than the twenty-dollar work-box; while as to the minister, these costly presents are counted as cash, as that much of a salary, when he is not able to make use of them. So these fancy, costly presents to ministers' families are money thrown away by the parishioner, and money lost to the persons intended to be benefited by them.

It is not in human nature to resist the mollifying influences of gifts: the great mind of Lord Bacon could not resist it; hence it is every where ground for impeachment and removal as to our judges, and there is a universal sentiment of approval, even in the midst of the most uncultivated of our population. In short, there is only one true, honorable, safe and manly course to be pursued in reference to our clergy, and if it were universally adopted, it would throw over this nation a conservative and an elevating influence which would make it in reality the glory of the whole earth; and it is this: Let every evangelical clergyman of all sects have a salary fully adequate to a plain, unostentatious mode of life, and let it be promptly, regularly and fully paid in money, to the very last cent; and never let him be presented with a single dollar or its value, except under circumstances which would effectually prevent the giver being known, except to that giver and to God, who will reward him openly in due time.

To pay a laborer half-a-dollar for a day's work, when it is acknowledged to be worth a dollar, and then to make a present to him of the other half—what is it? Your minister ought not to be treated thus. The laborer is worthy of his hire. A clergyman's salary is his right, it is for work done, and he is entitled to his pay in money, in full, and promptly at the day. But to seek to pay a part of it as a gratuity, is nothing less than a trickery, a subterfuge, a meanness, and a degradation, and is direct temptation, a bribe and a command: "Speak unto us smooth things." Hence these things are a profanation, and always will bring leanness to priest and people.

Since the above was put in type, it is stated that, "At one of the recent festivals in Boston, Judge Thomas of the Supreme Court, in urging the necessity and justice of giving an adequate support to clergymen, condemned donation-visits, as being mortifying confessions of the failure to pay an honest salary to the clergy, and as eking out an insufficient support by giving what is not wanted, and what is worse, making a charity of the wages of honest labor."

